# Overcoming Multidrug Resistance of Antibiotics via Nanodelivery Systems

**DOI:** 10.3390/pharmaceutics14030586

**Published:** 2022-03-08

**Authors:** Mohammad Imran, Saurav Kumar Jha, Nazeer Hasan, Areeba Insaf, Jitendra Shrestha, Jesus Shrestha, Hari Prasad Devkota, Salman Khan, Nisha Panth, Majid Ebrahimi Warkiani, Kamal Dua, Philip M. Hansbro, Keshav Raj Paudel, Yousuf Mohammed

**Affiliations:** 1 Department of Pharmaceutics, School of Pharmaceutical Education and Research, Jamia Hamdard, New Delhi 110062, India; mohammadimran2024@gmail.com (M.I.); nazeerhasan1994@gmail.com (N.H.); 2 Department of Biomedicine, Health and Life Convergence Sciences, Mokpo National University, Jeonnam 58554, Korea; Saurav.balhi@gmail.com; 3 Department of Pharmacognosy and Phytochemistry, Delhi Pharmaceutical Sciences and Research University, New Delhi 110017, India; ariba.insaf@gmail.com; 4 College of Pharmacy and Natural Medicine Research Institute, Mokpo National University, Jeonnam 58554, Korea; shresthasimon2011@gmail.com; 5 School of Biomedical Engineering, University of Technology Sydney, Sydney 2007, Australia; jesus.shrestha@student.uts.edu.au (J.S.); majid.warkiani@uts.edu.au (M.E.W.); 6 Graduate School of Pharmaceutical Sciences, Kumamoto University, 5-1 Oe-honmachi, Chuo ku, Kumamoto 862-0973, Japan; devkotah@kumamoto-u.ac.jp; 7 Department of Public Health, School of Allied Health Sciences, Noida International University, Greater Noida 203201, India; salmantomar7860@gmail.com; 8 Centre of Inflammation, Centenary Institute and University of Technology Sydney, Faculty of Science, School of Life Sciences, Sydney 2007, Australia; n.panth@centenary.org.au (N.P.); kamal.dua@uts.edu.au (K.D.); 9 Institute for Biomedical Materials and Devices, Faculty of Science, University of Technology Sydney, Sydney 2007, Australia; 10 Discipline of Pharmacy, Graduate School of Health, University of Technology, Sydney 2007, Australia; 11 Faculty of Health, Australian Research Centre in Complementary & Integrative Medicine, University of Technology Sydney, Ultimo 2007, Australia; 12 Therapeutics Research Group, The University of Queensland Diamantina Institute, Faculty of Medicine, University of Queensland, Brisbane 4102, Australia

**Keywords:** antimicrobial resistance, drug delivery, resistance mechanism, microfluidics, nanoparticles, lipid-based nanocarriers

## Abstract

Antibiotic resistance has become a threat to microbial therapies nowadays. The conventional approaches possess several limitations to combat microbial infections. Therefore, to overcome such complications, novel drug delivery systems have gained pharmaceutical scientists’ interest. Significant findings have validated the effectiveness of novel drug delivery systems such as polymeric nanoparticles, liposomes, metallic nanoparticles, dendrimers, and lipid-based nanoparticles against severe microbial infections and combating antimicrobial resistance. This review article comprises the specific mechanism of antibiotic resistance development in bacteria. In addition, the manuscript incorporated the advanced nanotechnological approaches with their mechanisms, including interaction with the bacterial cell wall, inhibition of biofilm formations, activation of innate and adaptive host immune response, generation of reactive oxygen species, and induction of intracellular effect to fight against antibiotic resistance. A section of this article demonstrated the findings related to the development of delivery systems. Lastly, the role of microfluidics in fighting antimicrobial resistance has been discussed. Overall, this review article is an amalgamation of various strategies to study the role of novel approaches and their mechanism to fight against the resistance developed to the antimicrobial therapies.

## 1. Introduction

Utilizing nanoparticulate materials to treat infectious diseases has been the subject of great interest in recent times. Infection resulting from multidrug resistance organisms (MDROs) are among the significant causes of morbidity and mortality worldwide [1]. In addition, antibiotics available for the treatment of MDROs are limited. Nevertheless, the development of new antibiotics to tackle MDROs infection requires huge economic and personnel investment and is laborious. Therefore, these critical clinical challenges emphasize the development of alternative and effective antimicrobial strategies to treat MDROs infections. Notably, owing to their unique physiochemical attributes, nanoparticles demonstrate therapeutic promise. Nanoparticles could be categorized in two ways: organic (e.g., lipid-based nanoparticle) or inorganic (e.g., nanoparticles of metals), and these have proved highly efficacious in treating various health complications [2]. Notably, organic nanoparticles have been widely reported to increase the bioavailability of drugs, enhance penetration and drug delivery, and improve antibacterial activities. The most attractive aspect of nanoparticles is the ability to incorporate different types of therapeutics, whether attached to their surface or incorporated within the structure; therefore, increasing the availability of drugs to the site of the action and better efficacy. In addition, nanomaterials can disrupt the bacterial cell membrane and target intracellular components and inhibit the proper functioning of the cellular machinery of microbes.

In 2008, the American Society of Infectious Diseases coined the term ESKAPE to categorize the deadly bacterial pathogens growing at pace and adapting to be multidrug-resistant against different antibiotics. The pathogens included *Enterococcus*, *Staphylococcus*, *Klebsiella,* and *Pseudomonas* species. The “escape” mechanism and behavior of these pathogens from the biocidal effect of contemporary marketed therapeutics causes life-threatening serious complications such as hospital-acquired infections [3]. Macrophages are responsible for eradicating foreign microbes, pathogens, and particles from the systemic circulation via a specific pathway. These phagocytic pathways could be exploited for the delivery of nanocarriers containing antibiotics for superior therapeutic effects. These pathways enable nanoparticles to be delivered directly to macrophages. It is believed that antibiotic-loaded nanocarriers can passively accumulate via the specific identification mechanism and uptake by phagocytes. Subsequently, the uptake of nanocarriers by phagocytosis leads to the release of drug payload in the infected cells, which eventually increases the permeation and exhibits superior therapeutic effects.

Moreover, biofilm-associated antimicrobial resistance is another concern. Therefore, nanocarriers have to play a role in overcoming these hurdles for better efficacy because they act as a protective coat, shielding against interactions, reducing the inactivation of drugs by biofilm and resident enzymes. In this scenario, nano-based drug delivery systems are promising for the treatment of either intracellular or biofilm-forming pathogenies infections. Diverse nanoformulations, such as liposomes and polymeric nanoparticles (NPs), have been developed and employed to deliver antibiotics to difficult-to-treat bacteria [3].

Therefore, the application of nanoparticles in antibacterial therapy to overcome MDR is an emerging approach. This review will provide insight into the mechanism of nanoparticles′ antibacterial activity and ability to overcome MDROs. In addition, the enhanced interaction of nanoparticles with antibiotics, microbial variation strategies to nanoparticles, challenges, and future prospects will be broadly discussed.

## 2. Antibiotic Resistance Mechanism in Bacteria

**Figure 1:**
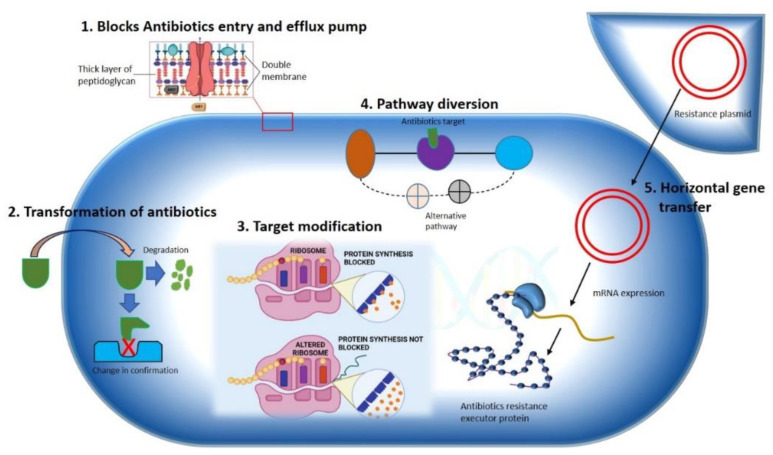
Mechanisms illustrating antibiotics resistance strategy used by bacteria. A Gram-negative bacterium is depicted various mechanisms for antibiotics resistance approaches against different antimicrobial agents are specified. (**1**) Hindering of antibiotics’ entry and removal using efflux pump. (**2**) Transformation of drug either by degradation or by changing drugs’ original confirmation. (**3**) Modification of antibiotics’ target by gene mutation. (**4**) Diversion of pathway containing drug target. (**5**) Horizontal transfer of gene.

The development of antibiotic resistance in pathologically lethal microorganisms is considered one of the foremost public health challenges to tackle infectious diseases worldwide. Nosocomial infections by multidrug resistance microorganisms increase the risk of life-threatening conditions to the patient in postoperative wards, burn units, and critical care units. For instance, hospitals are the major MDROs colonizing spot [4], but these are not limited to the hospital settings only. Community sites such as animal farms, biohazardous material dumping areas, freshwater environments, etc. [5,6] are a significant breeding ground for MDROs. The lack of aseptic techniques used in patient care, such as the use of one nonsterilized stethoscope or thermometer, ungloved or single-gloved hands in multiple patients in hospital wards, unethical and improper use of antibiotics in animal farms, dumping of unsterilized hospital waste in dumping sites and freshwater sources are increasing the risk of the spread of MDROs, in community environments. In this way, the horizontal transfer of resistant genes into surrounding microorganisms is also hastened. According to the nature of microorganisms, several mechanisms can be adopted to develop resistance toward specific antibiotics. In this topic, we will discuss some of the mechanisms adopted by bacteria for the development of resistance against different antibiotics (Figure 1).

### 2.1. Constrained Antibiotic Entry and Efflux Pumps

Phylogenic analysis of membrane protein signature sequence demonstrates that Gram-negative (diderms) bacteria possess bilayer membrane (outer membrane and inner membrane coverings) while another division of organisms exists with simple monolayer covering (monoderms) [7]. The outer membrane of Gram-negative bacteria contains some classical porins that are tightly regulated by certain genes and can be influenced by environmental factors [7], allowing minute molecules like amino acids and saccharides to pass through [8]. To access the specific target, this complex outer membrane of Gram-negative bacteria must be passed by most the antibacterial agents, for example, utilizing diffusion mechanism hydrophobic drugs can make an entry into the bacterial cell, hydrophilic antibiotics such as β-lactam pass through porins, while vancomycin gets hindered due to its structure. On the other hand, Gram-positive bacteria lack this complex membrane structure, making Gram-negative bacteria more antibiotic resistant than Gram-positive due to their outer membrane′s selective permeability [9]. Bacterial species that lack targets for specific antibiotics are naturally resistant to that antibiotic class. Some bacteria are naturally lacking in the targets of some antibiotics groups, which makes them intrinsically resistant to these antibiotics. *Mycoplasma* spp., for example, has a cell wall resistant to the cell wall synthesis inhibitor antibiotics, such as β-lactam and glycopeptides [10].

A bacterial efflux pump is another crucial mechanism for antibiotic resistance. The efflux pumps are found naturally in some bacteria or can be obtained from external sources. In bacteria, five genes that encode different efflux pumps are the major facilitator superfamily (MFS), ABC (ATP binding cassette) MDR transporter, resistance nodulation division (RND) family, staphylococcal multiresistance (SMR), and multidrug and toxic compound extrusion (MATE) families [11]. RND family proteins are found in the Gram-negative enteric bacteria *E. coli* and include inner membrane transporter efflux (e.g., AcrB), outer membrane protein channel (e.g., TolC), and a periplasmic accessory protein (e.g., AcrA). Numerous bacterial species such as *E. coli*, *P. aeruginosa*, *Campylobacter jejuni*, and *Neisseria gonorrhoeae* share a high homologous efflux pump such as AcrB/AcrB, mexB/MexB, B/CmeB, and mtrD/Mtrd [12]. Gram-positive bacteria, on the other hand, have only two types of efflux pumps (PmrA and NorA), both are present in *S. aureus* and *S. pneumoniae* and belong to the MFS family. Another MATE MDR efflux pump has been found in Gram-positive and Gram-negative bacteria. It uses PMF and the sodium ion gradient as an energy source, enabling the organism to develop resistance to a variety of antibiotics. Tetracycline resistance genes (63 tet genes reported) are the most common acquired resistance genes in both Gram-positive and Gram-negative clinical isolates, which encodes numerous efflux transporters [13]. Tetracycline resistance gene tet(63), which serves as an efflux pump, has been found in a multiresistance plasmid from *S. aureus* chicken isolates [14].

### 2.2. Transformation or Termination of Antibiotics

The modification and termination of antibiotics either by chemical alteration or molecular destruction is the most common approach used by bacteria to render antibiotics, making them futile with the use of enzymes. The enzymes involved in drug modification are categorized into three primary groups based on their reaction mechanisms: hydrolases, transferases, and oxidoreductases. β-lactamase is the important member of the group hydrolases, which catalyzes the hydrolytic breakdown of β-lactam ring present in cell wall synthesis inhibitor antibiotics groups such as penicillins, cephalosporins, carbapenems, monobactams, and clavulanate. A total of four different types of β-lactamase enzymes have been reported. Among them, extended-spectrum β-lactamase is the most potent enzyme which can inactivate the β-lactam antibiotics, including all penicillins, third-generation cephalosporins, and monobactam aztreonam [15] which became a challenge for the treatment of nosocomial infections [16]. Aminoglycoside-modifying enzymes (AMEs) are transferase enzymes that are found in most mobile genetic elements (MGEs), that facilitate the rapid spreading of genes. The clinically relevant member of the AMEs family is N-acetyltransferase, which acetylates aminoglycosides rather than O-phosphotransferases and O-adenylyltransferases, which adenylate and phosphorylate aminoglycosides in several locations, rendering antibiotics ineffective to its targets [17]. These antibiotic resistance genes are naturally expressed in antibiotic producing organisms such as species of *Streptomyces* like *S. griseus*, which converts streptomycin into inactive precursor streptomycin -6-phosphate. The presence of such genes in antibiotic producing organisms is still up for dispute as to whether they play a role in antibiotic resistance or only serve as housekeeping genes [18]. However, studies of these genes and their pattern could be helpful for the investigation of genetic information presents in MGEs found in various hospital isolates contributing modification of different lifesaving antibiotics.

### 2.3. Pathway Alteration to Avoid the Antibiotic Target Site

**Figure 2:**
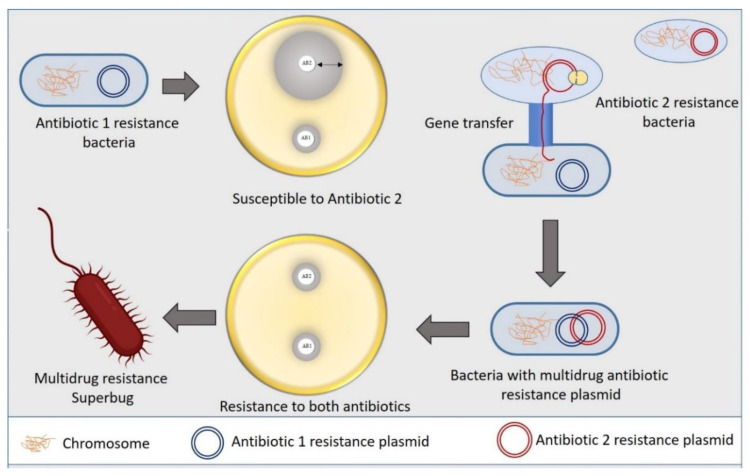
Mechanism of bacterial superbug formation by acquiring multiple drug resistance plasmid through horizontal gene transfer.

Bacteria commonly exploit alteration or skipping of the drug target to acquire resistance to specific antibiotics. Bacteria can evolve new metabolic sites that perform the same biochemical functions as the original target but are not inhibited by medications that target the original target. Antibiotics in the sulfa group target the bacterial folic acid synthesis pathway, which is missing in humans and higher eukaryotes. Eukaryotes get their folic acid from external sources, and they have a folate uptake system that most prokaryotes lack. Sulphonamide antibiotics primarily block dihydropteroate synthase (DHPS), a folic acid biosynthesis pathway enzyme absent in eukaryotes. Clinical isolates of some gut bacteria that have acquired a resistant variant of the DPHS gene by horizontal gene transfer have been reported to be resistant to sulphonamides (Figure 2). A unique DHPS enzyme variation generated from the genes *sul1* and *sul2* have been discovered in most sulphonamide-resistant enteric bacteria, which is sulphonamide insensitive but has equal binding efficacy with its substrate p-aminobenzoic acid [19,20]. This sulphonamide resistance mechanism is an excellent example of a pathway bypass resistant mechanism that avoids the antibiotic target. Other examples of pathway alteration mechanisms are the acquisition of external penicillin-binding protein (PBP2a) in MRSA clinical isolates and cluster van-genes in vancomycin-resistant enterococci. In both instances, the acquired modified enzymes functioned normally for cell wall synthesis even in the presence of cell wall synthesis inhibitor antibiotics. The mecA gene in *S. aureus*, which was probably obtained from *Sthaphylococcus sciuri*, encodes an alternative PBP2a enzyme that has a poor affinity for most β-lactam antibiotics, such as penicillins, cephalosporins, and carbapenems [21]. The acquired gene mecA placed into a large MGE gene cassette, [22], providing an alternative way to bypass the target of all β-lactam antibiotics, making the organism clinically more virulent than the wild type. The van genes (vanA, vanB, vanC, vanD, vanE, vanF, vanG, vanL, vanM, and van N) encode a biochemical system in enterococci that synthesizes D-Ala rather than D-Lac or D-serine (low resistance) and destroys the wild type “D-Ala-D-Ala” ending of peptidoglycan precursor molecules, lowering vancomycin affinity by thousands of times. A clinically contagious vancomycin resistant enterococci strain can be developed by acquiring a single cluster of van gene.

Bacteria can also adapt an alternative metabolic mechanism termed as a metabolic adaptation to resist some drugs. By metabolic adaptation, the daptomycin challenge in *S. aureus* redirects carbon flow from the TCA cycle into the pentose phosphate pathway, increasing the essential intermediates required for peptidoglycan biosynthesis intermediates, teichoic acids, and nucleosides, resulting in the development of a daptomycin non-susceptible strain from a susceptible strain [23]. Furthermore, when a daptomycin sensitive strain of *S. mitis* was exposed to daptomycin, the glucose metabolic pathway was transformed, resulting in the formation of a new daptomycin nonsusceptible strain [24]. The development of an alternative metabolic pathway to survive in a hostile environment containing antibiotics and the immune system of the host, makes bacteria more virulent.

### 2.4. Antibiotics Target Modification

Bacteria gain resistance to a wide spectrum of antibiotics by modifying target enzymes, either through genetic mutations that encode target enzymes or by modifying enzyme confirmations. Most bacteria acquire point mutations for genetic alteration, while phosphorylation, acetylation, and adenylation are the most common ways of modulating enzymatic confirmation. In both scenarios, a modified enzyme is formed that seems to have a low affinity for antibiotics while maintaining a wild-type affinity for its substrate. Rifamycin (RIF) antibiotics target DNA-dependent RNA polymerase by binding in the β-subunit RIF binding pocket, obstructing the pathway of nascent RNA. One step point mutation in the ropB (RNA polymerase) gene results in mutational resistance to RIF antibiotics. A single-step mutation substitutes one amino acid, decreasing drug affinity while maintaining the enzyme′s polymerase catalytic activity, allowing for RNA production similar to that of the wild type [25,26].

Another mechanism of bacteria to confer target modification is by enzymatic reform of drug target. The illustration of enzymatic alteration of the antibiotic target is best implied in macrolides resistance accomplished by the methylation genes to the target of macrolides, 23S rRNA ribosomal subunit [27]. The erythromycin ribosomal methylation (*erm*) gene produces adenine methylation at the A2058 position of the 50S ribosomal subunit′s 23S rRNA, V domain. More than 30 types of *erm* genes have been identified in MGEs, which may be responsible for the distribution of *erm* genes among different types of aerobic, anaerobic, and both Gram-positive and Gram-negative bacterial populations. Similarly, cfr genes encoding a wide range of methylase family members have been found in *S. aureus*, *E. feacalis*, *E. faceium*, and a few Gram-negative isolates that have been integrated into MGEs plasmids, conferring resistance to phenicols, lincosamides, and streptogramin-A. [28].

## 3. Mechanism of Advanced Nanotechnological Approaches against Antibiotic Resistance

**Figure 3:**
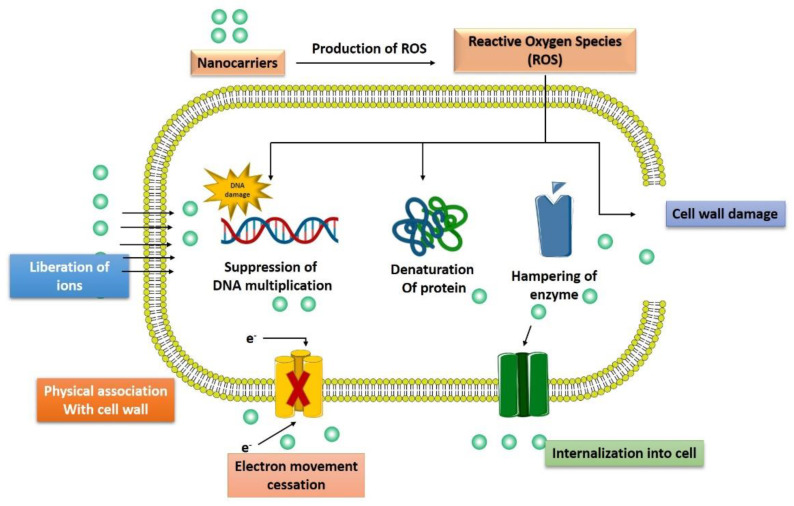
Multiple pathways followed by advanced nanotechnological approaches to combat the antibiotic resistance.

Recently, various drug-loaded nanoparticles have made remarkable progress with their promising antibacterial activity in a wide range of bacteria. They offer greater protection and combat the multidrug resistance against these micro-organisms. For example, silver nanoparticles (Ag-NPs) exert robust, broad-spectrum antibacterial efficacy by generating reactive oxygen species (ROS) to kill bacteria [29]. In the following section, we have discussed various mechanisms/pathways targeted by nanoparticles to control bacterial growth or exert bactericidal activity (Figure 3).

### 3.1. Interaction with the Bacterial Cell Wall

The bacterial cell wall is a crucial defensive barrier that allows bacterial to resist external insults and maintain their natural morphology. Among various components of the cell wall, lipopolysaccharide (LPS) is particularly found on Gram-negative bacterial, while teichoic acid is found in Gram-positive bacteria [30]. Comparatively, nanoparticles are found to be more efficacious against Gram-positive than Gram-negative bacteria because LPS and phospholipid in Gram-negative bacteria form a barrier against penetration of nanoparticles and macromolecules while teichoic acid and peptidoglycan along with numerous pores in Gram-positive allows entrance of foreign particles including nanoparticles/macromolecules [31]. A novel nanoparticles of hydroxyapatite whisker/nano zinc oxide was synthesized by Yu and associates demonstrated the better antimicrobial effect of these nanoparticles on different types of bacteria such as *S. mutans*, *Candida albicans*, and *S. aureus* compared to *E. coli*. The bactericidal activity of nanoparticles was dependent on (1) the composition of a bacterial cell that might results in better activity against Gram-positive bacteria; (2) the specific parts of Gram-negative bacteria (for example LPS) that stop the binding of nanoparticles to the bacterial cell wall and modulate the endo and exocytosis of ions from bacterial cell membrane. (3) the dimensions of the bacterial cell wall (20–80 nm in Gram-positive and 1.5–10 nm in Gram-negative bacteria) affect the biological activity of nanoparticles [32]. Another study observed that phospholipid groups in the LPS layer of *E. coli* (Gram-negative) intermingle with ε-poly-l-lysine mediated by electrostatic attraction, resulting in the damage of the cell membrane. Nevertheless, in the case of *Listeria innocua* (Gram-positive), the film consists of lysine-derived phospholipids which is amphoteric in nature. Therefore, it lacks enough negative charge to attract cationic peptides. Thus, the cell membrane of *L. innocua* has lower permeability than that of *E. coli* [33]. Another study investigating the antibacterial potency of nanodiamonds found that these nanodiamonds form covalent bonds with proteins and other molecules on bacterial cell walls. The binding of nanodiamonds with bacterial intracellular elements could further impair the crucial enzymes and proteins, ultimately hindering the bacterial metabolism leading to cell death [34].

### 3.2. Inhibition of Biofilm Formations

Bacterial biofilm is a matrix of extracellular polymeric components around bacteria colonies that renders them resistant to antibiotic therapy, chemical disinfection, immune response [35]. Nanoparticles such as zinc oxide nanoparticles (ZnO NPs) are already established as a potent antibiofilm formulation. Kaur et al., 2020 showed that ZnO NPs inhibit biofilm in the range of bacteria and alter the bacterial cell membrane permeability [36]. Similarly, synthetic drugs or natural compound-loaded nanoparticles are potent inhibitors of bacterial biofilm. Curcumin-loaded chitosan nanoparticles exhibited robust antibiofilm activity against *Candida albicans* and *Staphylococcus aureus*. This nanoparticle not only reduced the thickness of biofilm but also kills the bacteria [37]. Similarly, synthetic antibiotics such as ciprofloxacin-loaded polylactic acid-glycolic acid (PLGA) nanospheres [38], azithromycin-loaded PAMAM-AZM nanoparticles [39], cefotaxime-loaded chitosan nanocarriers [40] can reduce >95% of *Pseudomonas aeruginosa* biofilm while roxithromycin-loaded cyclodextrin nanocarriers [41] and trifluorosan-loaded micellar nanocarriers [42] and vancomycin-loaded PGLA nanospheres [43] significantly inhibited the *Staphylococcus aureus* biofilm resulting in bacterial death.

### 3.3. Activation of Innate as Well as Adaptive Host Immune Response

Various investigations have revealed the interaction of nanoparticle formulation with the innate immune system leading to immune activation, and these are mediated by immune cells such as monocytes, macrophages, neutrophils, and other cells that express pattern recognition receptors (PRR). These cells release several cytokines and chemokines as a defensive mechanism against invading pathogens, including bacteria [44]. Some nanoformulation can amplify the PRR-driven inflammation, for example, the production of interleukin-1β (IL-1β), while another nanoformulation amplifies the effects of the bacterial stimulation. IL-1 signaling triggered by *Streptococcus suis* serotype 2 leads to the clearance of bacteria and inflammation of systemic disease as revealed by the mice model of infection [45]. Some bacterial strain for example, *Staphylococcus aureus* have developed an immune system bypass mechanism by releasing biofilms that lead to a significant reduction in the detection of any immune response [46]. The host innate immune system activation in response to bacterial infection primarily occurs via the identification of a pattern of bacteria surface by PRRs, such as toll-like receptors. Moreover, microorganisms that invade the cell are identified by a family of cytosolic PRRs called NOD-like receptors (NLRs). The pivotal function of the host innate immune response is to halt bacterial development and spread. In contrast, impairment of immune responses, for example, chronic wounds of diabetes mellitus patients, provides a favorable environment for bacterial for excessive proliferation leading to delay in wound healing and further tissue damage/infection [47]. Nanoparticles can impact the innate immune system response against bacteria in various ways, such as (1) nanoparticles coating the bacterial surface can result in inhibition of cellular uptake of bacteria; (2) nanoparticles (less than 30 nm size) coating bacteria can also conceal surface pathogen-associated molecular patterns (PAMPs) and activate PRR; (3) nanoparticles may compete with the process of phagocytosis of bacteria; (4) nanoparticles may induce alteration in the epigenetic pathway and (5) cytotoxic nanoparticles result in altered cell membrane permeability that becomes favorable for bacterial influx. Nanoparticles coating bacterial surfaces can serve multiple purposes, such as inhibition of bacterial pathogenicity, invasion, and concealing the PAMPs to which TLRs or NLRs would otherwise bind [48]. The pretreatment of mice macrophage with gold or silica nanoparticles can inhibit phagocytosis of killed *E. coli* [49], while superparamagnetic iron oxide nanoparticles can inhibit the uptake of killed *S. pneumoniae* by macrophage derived from bone marrow [50]. Likewise, pretreatment with gold nanoparticles to human monocytes reduces the immune activity of monocytes to live Bacilli Calmette-Guérin as seen by the level of cytokine production [51].

### 3.4. Generation of Reactive Oxygen Species

The toxic effect caused by various nanoparticles, such as silver nanoparticles, is due to the generation of ROS, impairment in antioxidant enzyme level, and production of free radicals, such as superoxide anions, hydrogen peroxide, hydroxyl radical, singlet oxygen, and hypochlorous acid [52]. Oxidative stress is harmful to both bacteria as well as the human body as it results in the progression of various diseases [53]. Ag-NPs can provide an alternative approach for multidrug-resistant bacterial strains such as *S. aureus* and *P. aeruginosa*. A study on these two bacterial strains isolated from breast inflamed goats found that Ag-NPs exert promising antibacterial activity via production of ROS, malondialdehyde (a marker of oxidative stress), and loss of essential components such as proteins and sugars due to leakage in the cells of the bacteria. The Ag-NPs also downregulated the initiation of antioxidant enzyme; glutathione, superoxide dismutase, and catalase [54]. Similarly, zinc oxide nanoparticle (ZnO NPs) is another promising formulation that results in an oxidative stress-mediated genotoxic effect in a radiation-resistant bacterial strain, *Deinococcus radiodurans*. A study by Singh et al., 2020 demonstrated that ZnO NPs were internalized remarkably inside the *D. radiodurans* and these nanoparticles initiate significant ROS generation, oxidation of protein molecules, and DNA impairment along with depletion in the thiol levels [55]. Similarly, copper-maleamate-functionalized mesoporous silica nanoparticles and cadmium oxide nanoparticles [56] were also found to possess potent antibacterial activity mediated by induction of oxidative stress.

### 3.5. Induction of Intracellular Effects

Nanoparticles can interfere with a bacterial intracellular function such as protein synthesis, enzyme function, or impair the genetic material and kill them. ZnO NPs were found to reduce the expression of various DNA overhaul genes (Mut S, Mut L, Ung, Mut M, DNA polymerase, and DNA ligase) and metabolic pathway genes (aconitase, succinate dehydrogenase) and increase the expression of DNA impairment response genes (Ddr A, Ddr B, Ddr D) [55]. Carbonate-coated Ag-NPs bind to the *E. coli* proteins such as tryptophanase and results in loss of enzyme activity [57]. Silver (Ag) ions are recognized to bind to the DNA of bacteria and it was revealed that these silver ions exposed to *E. coli* and *S. aureus* result in condensed DNA, ultimately arresting bacterial multiplication [58]. The interaction of Ag-NPs to *E. coli* also results in the upregulation of 161 genes and downregulation of 27 genes [59]. The upregulated genes were associated with a number of functions such as the citric acid cycle (sdhC), protein efflux (fsr, yajR, emrE), membrane structure and biofilm formation (bolA), electron transfer (sdhC), cellular transport (mdfA), and DNA repair (recN, uvrA, ybfE, yebG, ssb, sbmc, and nfo) [60].

## 4. Nanotechnology to Overcome Antimicrobial Resistance

**Figure 4:**
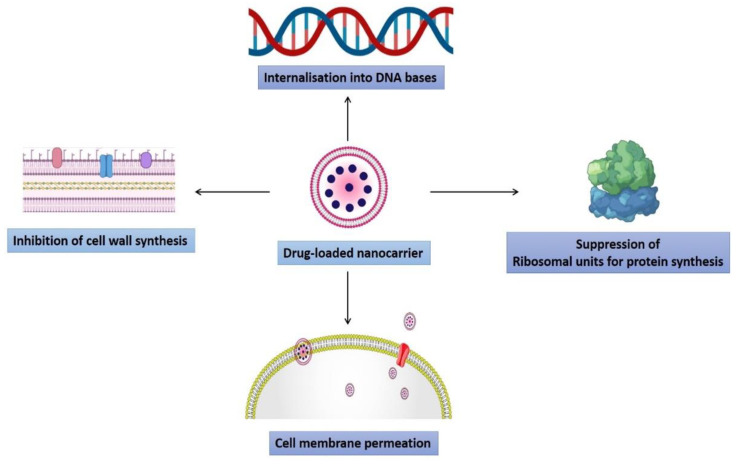
The illustration shows the role of drug-loaded nanocarrier in inhibiting the antibiotic resistance by internalizing into the DNA bases, inhibiting cell wall synthesis, suppressing ribosomal units, and by increasing the permeation through cell membrane.

Generally, nanotechnology comprises all systems whose mechanical, chemical, and pharmacological effects are modified to become substantially distinct, leading to special procedures due to the nanosized array of particles [61,62,63,64]. Nanoparticles utilized as drug delivery carriers are typically 100 nm in at least one dimension and comprise disintegrating components such as organic polymers, lipids, and metals [65,66,67,68]. However, the rapid bacterial antibiotic resistance development, along with slower and declining drug development, as a result, there is a race between medicinal progress and the establishment of bacterial drug resistance [69]. In modern medicine, conventional antibiotics impose various drawbacks; low bioavailability, little penetration into the infection nidus, and the formation of drug-resistant microorganisms are only a few examples. Nanoparticles can preserve drugs from enzymatic breakdown and extend drug release, improving half-life and bioavailability [70]. Most MDR infections necessitate prolonged antibiotic therapy, as well as tissue excision (i.e., surgical removal) in some situations, resulting in poor treatment compliance and high healthcare costs [71]. NPs are feasible alternatives to traditional antibiotics due to a variety of features. For example, the massive surface-area-to-volume ratio enhances interfacial interaction with the targeted organism. These NPs can act as nanoscale biomolecules, engage with bacterial cells, influence cellular membranes invasion, and intervene with molecular mechanisms. Second, NPs may improve the restricting spread of antibiotic resistance [72]. Antibiotic-tagged nanomaterials allow for higher antibiotic concentrations near the location of infection and boost antibiotic bacterial binding [73]. These nanomaterials act by crossing the microbial cell membrane, producing change in the size, the shape of the bacterial membrane and altering the metabolic pathways. Within the cells, NPs interface with the biological pathways, impede enzymes, deactivate proteins, give rise to oxidative stress, electrolytic imbalance, and alter genetic variants [74]. These nanoparticles can destroy pathogens primarily through the physical or metabolic process [75]. NPs could also bypass multidrug resistance mechanisms such as by significantly lowering absorption and enhanced outflow of drugs from microbial cell biofilm development and intracellular bacteria [76]. Various kinds of nanoparticles are present and act by different mechanisms, some of which have been thoroughly discussed concerning their role in antimicrobial resistance (Figure 4).

### 4.1. Polymeric Nanoparticles

Polymeric nanoparticles (NPs) are particles with diameters ranging from 1 to 1000 nm that can be loaded with active compounds encapsulated within or surface-adsorbed onto the polymeric core. These NPs have shown considerable promise for targeted medication delivery in treating various ailments [77]. Additionally, their nanostructures can be created using a diversity of organic or synthetic precursors such as collagen, chitosan, gelatin, or glycoprotein, as well as polyethylene glycol, polylactic acid, poly (lactic-co-glycolic acid) (PLGA), polylactic acid (PLA), or polycaprolactone (PCL) [78]. Enveloping chitosan into nanoparticles enhances the surface-to-volume ratio, resulting in enhanced surface charge density, increased antimicrobial activity, attachment to microorganisms′ cell walls and membranes becoming stronger and more prevalent [79]. High-molecular-weight chitosan nanoparticles are more effective against Gram-positive bacteria than Gram-negative bacteria. Chitosan nanostructures with a low molecular mass are more effective against Gram-negative bacteria than Gram-positive bacteria [76]. Caetano et al. studied a chitosan–alginate membrane healing effects on a cutaneous lesion in rats and discovered that the incision was not infected, fibroplasia grew significantly, fibroblasts were better distributed in the newly formed tissue, and scar tissue quality was improved. Furthermore, the inflammatory infiltration was considerably reduced on the seventh day of treatment with the chitosan–alginate membrane, followed by a drop in neutrophils and CD4+ cells, indicating the chitosan–alginate membrane may be superior at regulating the inflammatory stimuli [80]. The antibacterial and antifungal activity of uncoated and chitosan-coated Fe_3_O_4_ nanoparticles against five species, *E. coli*, *B. subtilis*, *C. albicans*, *A. niger,* and *F. solani*, was investigated by Nehra et al. The findings revealed that the particle size on average of Fe_3_O_4_ and chitosan-coated Fe_3_O_4_ NPs was reported to be 10.4 and 11.4 nm, respectively. The average inhibitory zone width of chitosan-coated Fe_3_O_4_ NPs was 14.5 to 18.5 mm. *F. solani/A.niger <C. albicans < E. coli/B. subtilis* were affected by chitosan-coated iron oxide nanoparticles (*p* > 0.001). Furthermore, E. coli showed stronger antibacterial action than *B. Subtilis* because the surface charge of Gram-negative bacteria is more negative than the charge of Gram-positive bacteria. Therefore, metal oxide NPs′ positively charged surface binds to the negatively charged membrane of the cell and cause bacterial cell disruption [81]. Piras et al. studied the chitosan nanoparticles (CS-NPs) loaded with the antimicrobial peptide temporin B (TB) and evaluated them in vitro for the long-term antimicrobial property against clinical isolates of *Staphylococcus epidermidis*. They reported an increased encapsulation efficiency of formulation up to 75%. In the experimental settings used, kinetic release measurements revealed a peptide release from the nanocarrier in a linear fashion. Surprisingly, encapsulating TB in CS-NPs reduced the peptide′s cytotoxicity against mammalian cells substantially. Furthermore, for at least four days, the nanocarrier demonstrated prolonged antibacterial activity against diverse strains of *Staphylococcus epidermidis*. At the end of the study, the number of live bacteria was reduced by up to four logs compared to ordinary CS-NPs. The constant release of TB decreased the potentially viable bacterial count even more, eliminating residual cell renewal and guaranteeing a long-lasting antibacterial impact [82].

### 4.2. Liposomes

Liposomes were discovered in the mid-1960s [83]. They are made up of a bilayer membrane made of amphipathic phospholipids that envelop an interior area that has been widely researched for antimicrobial drug delivery [84]. Liposomes are biocompatible, biodegradable, and innocuous vesicles capable of encapsulating and transporting both hydrophilic and lipophilic medicines, allowing for precise medication release at the site of infection [83,85]. The use of lipid vesicles as drug carriers considerably impacts drug distribution and reduces severe side effects during antibiotic therapy [86]. Woudenberg et al. established that once a day, liposomal ciprofloxacin achieved the same results as the free medicine administered twice daily against rats′ *Klebsiella pneumoniae* and *Pseudomonas aeruginosa*. Liposomal ciprofloxacin showed delayed elimination and concentrations in the blood and tissues that are increased and persistent. At high doses, PEG-coated liposomal ciprofloxacin proved harmless. Additionally, they established ciprofloxacin′s enhanced efficacy in the treatment of *Klebsiella pneumoniae* when delivered in PEG-coated liposomes rats with pneumonia [87]. Liu et al. created antibacterial peptide-modified azithromycin-loaded liposomes (AZT-LPs) against methicillin-resistant *Staphylococcus aureus*. They discovered that DP7-C injected into the LP bilayer membrane served as a carrier to encapsulate the antibiotic AZT and synergized the antibacterial effect of the entrapped AZT. They also showed that the MIC values of AZT-LPs were marginally lower than those of free AZT in an in vitro antibacterial investigation. In contrast, an in vivo antibacterial analysis revealed that D-LPs had a powerful antibacterial impact and demonstrated substantial synergistic effects with AZT, significantly augmenting AZT′s antibacterial activity [88].

### 4.3. Solid Lipid Nanoparticles

Solid lipid nanoparticles (SLNs) are nanoparticles comprised of solid lipids stabilized by an emulsifying layer in aqueous dispersion. They are similar to nanoemulsions in which the inner liquid lipid is replaced with a solid lipid [89]. These are a type of lipid-based vesicular structure that has received popularity because of their capacity to deliver drugs at a controlled and site-specific rate [90]. Additionally, surfactants are employed in emulsification to improve SLN stability [91]. They are biopolymers of natural or synthetic origin and are suitable for encapsulating lipophilic drugs [92]. SLNs containing antitubercular prepared using microemulsion technique drugs showed twofold inhibition of *Mycobacterium marinum* compared to pure antitubercular drugs [93]. Jalal et al. reported an improvement in antibacterial function of rifampicin-loaded SLNs, against *B. abortus*. They found out that Rif-SLNs had twice the antibacterial activity of unbound rifampicin. Furthermore, due to drug encapsulation, Rif-SLNs limit inactivation and, when combined with a sustained release over time, work more quickly and effectively on bacteria than unbound rifampicin [94]. Singh et al. established the cefuroxime axetil-loaded SLNs (CA-SLNs) for enhanced antibacterial activity against bacterial biofilms. They reported a lower MIC value against *S. aureus* of CA-SLNs than the CA solution due to small size nanoparticles. The nanosize allows the drug to permeate the cells and effectively destroy the organism. According to the authors SLNs can improve the efficiency of CA against *S. aureus* bacterial biofilm [95]. Rahul et al. demonstrated vancomycin′s enhanced entrapment efficiency and antimicrobial activity in solid lipid particles by ion-pairing with linoleic acid. The main aim of their study is the coadministration of vancomycin with a fatty acid (FA) to improve entrapment efficiency and antibacterial activity at the same time. They established that VCM-LA2 conjugate imparted higher lipophilicity and increased antimicrobial action of VCM-LA2 SLNs was mostly due to the increased lipophilicity of the VCM-LA2 compound may have allowed for permeation into the bacterial cell membrane via SLNs, controlled delivery of VCM from SLNs due to ion linking with anionic LA, and LA and VCM forming a nano delivery mechanism composed of two antibacterial agents acting through different pathways [96]. Ghaffari et al. established that when compared to the free drug, the minimum inhibitory concentration (MIC) and minimum bacteriostatic concentration (MBC) of amikacin in SLNs could be around two times lower. As a result, fewer amikacin doses in SLNs can cure the condition mostly with fewer unintended negative consequences and more excellent reliability. Moreover, the small particle size of the intended SLNs may further improve drug diffusion into the bacterial cell [97].

### 4.4. Nanostructured Lipid Carriers

Nanostructured lipid carriers (NLCs) are the first-generation lipid nanocarrier, comprise liquid and solid lipid as a lipidic phase, whereas surfactant as an aqueous phase. These nanocarriers are slightly different from SLNs because they contain liquid lipid, which remains absent in the former [90,98]. Additionally, due to its unique structural orientation- disordered structures of these nanocarriers enable the load of the higher drug without the leakage of the drug throughout the systemic circulation, which has been the main issue reported with the SLNs and other nanocarriers [99,100]. Therefore, various NLCs formulations have been designed for antibacterial efficacy. These formulations containing antibacterial drugs or antibiotics exhibited superior therapeutic efficacy compared to conventional therapy. Moreover, such formulations demonstrated excellent efficacy against bacterial resistance, which makes the therapy superior. Hence, some formulations have been discussed in this section exhibiting the better efficacy of NLCs as compared to the conventional and control therapy [101]. Rita et al. designed NLCs for the delivery of natural antimicrobial agents such as plumbagin, eugenol, hydroquinone, and alpha-tocopherol. These nanocarriers were constructed using the ultrasonication method and characterized in terms of morphological evaluation, in vitro release, and in vitro antibacterial efficacy. The findings revealed the strong toxicity of drug-loaded NLCs towards *F. oxysproum* as compared to free drugs. Contrarily, the toxicity effects were reversed in the case of human cultured cells where drug-loaded NLCs exhibited less toxicity as compared to free drug treatment on the human culture cells. Vairo et al. developed two different drug-loaded nanosystems in which sodium colistimethate (SCM) and amikacin (AMK) were loaded separately and evaluated against the bacterial strain, specifically, *A. baumanii.* The outcomes of this study demonstrated the lower toxicity of drugs when loaded in NLCs compared to free drug treatment. The particle size of these two nanosystems varied from the range of 50–200 nm, whereas the zeta potential range was −10.0 mV to −25.0 mV. The in vivo findings revealed that the dosing interval and frequency could be reduced in the case of SCM-NLCs, also notably, the dose was reduced by almost tenfold in SCM-NLCs (6 mg/kg) to achieve similar therapeutic effects as compared to free SCM (60 mg/kg). Hence, the encapsulation of SCM in NLCs demonstrated an effective and superior efficacious approach against resistance developed by *A. baumanii* [102].

### 4.5. Dendrimers

Dendrimers are cornerstone nanostructures with specific design and low polydispersity that are layer by layer (in ′generations′) surrounding a core unit, resulting in a great amount of control over size, branching points, and surface functionality [103]. Polyamidoamine (PAMAM) dendrimers are a large majority often employed for the delivery of drugs [104], dendrimers′ highly branching structure affords tremendous surface area/size ratios, leading to substantial in vivo responsiveness to microbes [105]. The structures of both the dendrimer and the drug influence drug entrapment. As a result, it is critical to comprehend the chemistry behind the dendrimer design [104]. Chauhan et al. has demonstrated the increased solubility of resveratrol by forming a resveratrol-dendrimer complex. PAMAM dendrimers were used to improve resveratrol solubility in aqueous media to generate drug–dendrimer complexes and assess the stability and antioxidant activity of resveratrol dendrimer complex formulations. Additionally, PAMAM dendrimer significantly increased resveratrol′s water solubility; compared to resveratrol alone, the dendrimer–resveratrol combination was very stable [106]. Svenningsen et al. reported an increment in the antimicrobial activity when conjugated with a PAMAM dendrimer. Ciprofloxacin was conjugated to a DAB-core G0 PAMAM-dendrimer and tested for antibacterial activity against various clinically significant Gram-positive and Gram-negative bacteria. The conjugate had a favorable dendritic effect in both types of bacteria [107]. Bosnjakovic et al. reported sustained-release erythromycin (EM) by conjugating it with dendrimer of poly(amidoamine). An ester bond was exploited to attach EM to poly(amidoamine) dendrimer (PAMAM). Additionally, the cytotoxicity, effectiveness, and antibacterial characteristics of macrophages (RAW 264.7 cells) linked with inflammation around the prosthetic device were investigated. The conjugate was noncytotoxic and significantly reduced nitrite levels by 42% compared with untreated cells and free EM. However, compared to free EM, the conjugate′s inhibitory zone on bacterial growth at varied doses demonstrated comparable activity. Their findings revealed that dendrimer-EM conjugates had a high drug payload (16%), enhanced solubilization, and resulted in higher activity [108].

### 4.6. Inorganic Nanoparticles

**Figure 5:**
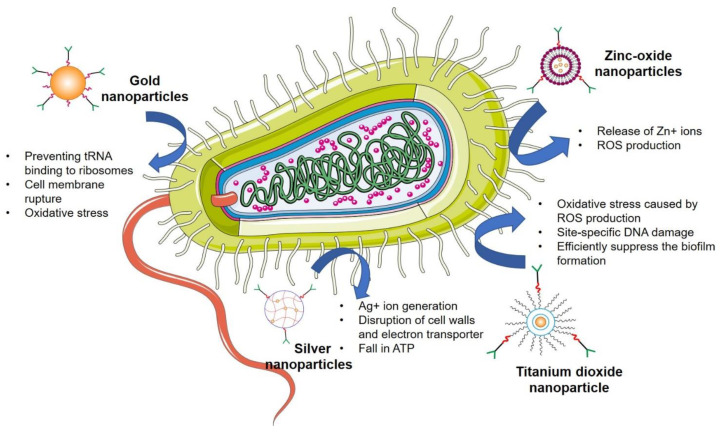
Inorganic nanoparticles and their mode of antibacterial mechanism.

Inorganic nanomaterials have been used for decades or are continuously being made as antimicrobials [73]. Because of their inherent antibacterial capabilities, inorganic NPs displayed improved effectiveness as nanobiocides as well as nanocarriers for antimicrobial medicines [109]. Different forms of inorganic nanoparticles have been created and classified depending on their constituents′ nature [110]. Moreover, various functions of inorganic nanomaterials against microbes have been reported, including photocatalytic generation of oxidative stress (ROS), which destroy cellular and viral major components, cause breakdown of the bacterial cell wall/membrane, disrupt energy transfer, and restrict enzymatic activity and nucleic acid synthesis [105]. This section investigates the many forms of inorganic nanoparticles and investigates the chemistry of their hazardous and/or nontoxic properties as well as antimicrobial methods of action (Figure 5) [111].

#### 4.6.1. Silver Nanoparticles

As a metal, silver has been used as a disinfectant for over 1200 years. It has been widely employed in the treatment of clinical disorders such as infant eye prevention, topical burn wounds, and orthopedic infections. Silver is a powerful antibacterial agent that kills many bacteria while being relatively nontoxic to mammalian cells [112]. Silver nanoparticles (AgNPs) are efficient against a broad range of pathogens because of their small size and vast surface area [77]. Isabelle et al. synthesized surface coating of polydopamine, which synergized the antimicrobial activity of silver nanoparticles (PDA-AgNPs). The findings revealed that PDA-AgNPs were superior antibacterial agents in fluorescence-based growth curve experiments on *E. coli.* The PDA-Ag interaction of PDA-AgNPs boosted ROS production and resulted in severe bacterial membrane damage [113]. Hamed et al. showed the synergism of silver with N-acetyl cysteine (NAC). NAC considerably increased Ag NPs′ antimicrobial efficacy against all pathogens studied. Moreover, a transmission electron microscope (TEM) investigation revealed the breakdown of cell walls in both bacteria and fungi. Ag NPs exhibited superior antibiotic activity, and NAC significantly improved their antimicrobial activity against MDR pathogens, providing a novel, safe, effective, and low-cost therapeutic method to reduce MDR pathogens prevalence [114].

#### 4.6.2. Gold Nanoparticles

Gold nanoparticles (Au-NPs) are projected to be particularly effective in the development of bacterial medications due to their nontoxicity, high functionalization capacity, polyvalent effects, simplicity of detection, and photothermal activity [115]. These NPs exhibited their antibacterial efficacy primarily through two mechanisms: first, by inhibiting ATPase activity, the membrane potential is reduced, resulting in a decrease in ATP level; second, by preventing NA from binding to ribosomes [116]. In one study, 5-fluorouracil-functionalized Au-NPs were evaluated for antibacterial and antifungal activity against *Micrococcus luteus*, *S. aureus*, *P. aeruginosa*, *E. coli*, *Aspergillus fumigates*, and *Aspergillus niger* by Tiwari et al. They exhibited these results due to their easier absorption, and these nanoparticles displayed higher pursuit on Gram-negative bacteria than Gram-positive bacteria [117]. Lima et al. discovered the antibacterial activity of Au-NPs (5 nm) against E. coli and Salmonella typhi (*S. typhi*) bacteria. Their findings revealed that these nanoparticles suppressed colonies of *E. coli* and *S. typhi* by 90–95%. The researchers found out that the roughness and the temperature were two elements that influenced the biocidal capabilities of the medium′s dispersion of Au-NPs [118].

#### 4.6.3. Zinc Oxide Nanoparticles

Zinc oxide nanoparticles (ZnO-NPs) have been shown to have a huge potential against microbes and reported being stable in preclinical studies [119]. ZnO NPs showed bactericidal efficacy against Gram-positive and Gram-negative bacteria, as well as high-temperature and pressure-resistant spores [115]. Such NPs, with a high surface-to-volume ratio, enable greater synergy with germs and consequently have superior antifungal and antibacterial properties [120]. Azam et al. proposed a comparison of the antibacterial activity of various metal oxides against Gram-positive and Gram-negative microorganisms. The finding of their study revealed that these NPs showed strong antibiotic potency, whereas Fe_2_O_3_ nanoparticles had the lowest bactericidal activity. The following antibacterial activity sequence was demonstrated: ZnO> CuO > Fe_2_O_3_ [121]. Xie et al. established the antibacterial efficacy of ZnO NPs compared to *Campylobacter jejuni*. They hypothesized that cell membrane rupture and oxidative stress in *C. jejuni* generated the antibacterial mechanism of ZnO NPs. Their findings indicated that these nanoparticles induced minor modifications, significant membrane permeability, and elevated oxidative stress gene expression (up to 52-fold) in *C. jejuni* [122].

#### 4.6.4. Titanium Dioxide Nanoparticles

Titanium dioxide nanoparticles (TiO_2_ NPs) are mass-produced worldwide for usage in a variety of applications [123]. These nanoparticles could be employed in food products and a constituent in various pharmaceutical and cosmetic products, such as sunscreens and toothpaste [124]. It is believed that oxidative stress caused by ROS production is the primary mechanism for TiO_2_ nanoparticles. These ROS are also responsible for causing site-specific DNA damage. Roy et al. investigated the impact of TiO_2_ nanoparticles combined with various medications on methicillin-resistant *Staphylococcus aureus* (MRSA). The antibacterial activity against MRSA of beta-lactams, cephalosporins, aminoglycosides, glycopeptides, macrolides, lincosamides, and tetracycline was boosted by TiO_2_ nanoparticles. In a further experiment, they discovered that the presence of TiO_2_ nanoparticles reduced MRSA′s antimicrobial resistance to several drugs [125]. The antifungal effect of TiO_2_ NPs on fungal biofilms (fluconazole-resistant *Candida albicans* standard strains) was investigated by Haghighi et al. As per their findings, the TiO_2_ NPs exhibited better antifungal efficacy on the fluconazole-resistant strain of *C. albicans* microflora. Findings revealed that these TiO_2_ NPs could efficiently suppress biofilms produced by fungi, particularly those produced on the medical device interface [126]. Apart from these findings, multiple developed nanoformulations against various microbial infections and antimicrobial resistance have been mentioned in Table 1.

## 5. Microfluidics for Combating Antimicrobial Resistance

Rapid and accurate detection of pathogenic bacteria and their antibiotic resistance profiles to identify the most effective antibiotic regimen against sepsis and life-threatening bacterial infections is crucial to decrease the mortality rate and treatment expenses [127]. However, the existing diagnosis and characterization techniques do not address this issue fully and require multiple growth steps, which is time-consuming and limited by low positive rate and inability to differentiate between strains and species of bacteria [128]. To overcome this challenge, alternative methods like matrix-assisted laser desorption/ionization-time of flight (MALDI-TOF) mass spectrometry (MS) are being used for earlier detection of pathogens with increased reproducibility and repeatability, but they cannot provide any information on antibiotic susceptibility and still require culture [129,130]. Recently, multiplex PCR assays have enabled rapid identification of organisms and only some specific antibiotic resistance profiles [131]. The delays in rapid and accurate diagnosis force clinicians to administer broad-spectrum antibiotics to save lives, which may not be the most effective antibiotic regimen, indirectly increasing antibiotic resistance [130].

Microfluidics is the science and technology that deals with the manipulation of fluids at a nanoliter scale in microchannels [132,133]. The fluid behavior in these microchannels is different from conventional flow due to the small scale of the system. Microfluidic chips are small portable chips containing microchannels, valves, reaction chambers, and sensors which can be applied for sample preparation, separation, reagent manipulation, bioreaction, and detection [128]. These chips consume a substantially low volume of samples and reagents, provide increased automation and parallelization, allowing accurate and high-throughput analysis of microbes at a significantly reduced cost and time [134]. The small size, portability, and reproducibility of these microfluidic chips complemented with the capability to be incorporated with biosensors and coupled with various detection techniques like MS, PCR, LAMP etc. makes them superior to the conventional methods for detection and analysis of microorganisms [135]. This will ultimately result in early diagnosis, initiation of the most effective treatment, decreased hospital stay and expense, and infection-associated morbidity and mortality.

**Figure 6:**
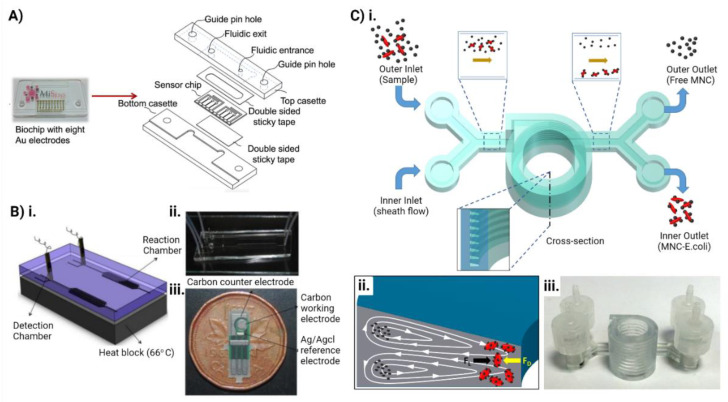
Microfluidic chips for microbiome purification, detection and downstream analysis. (**A**) Image of the custom designed MiSens biosensor with exploded view of the chip design. The fully automated microfluidic-based sensor was used for real-time bacteria detection. (Reproduced with permission from [139]). (**B)** Schematic illustration of the microfluidic chip for *E. coli* detection. (**i**) The PDMS chip contained two parallel microfluidic chips for DNA detection and negative control. The microfluidic chip composed of a reaction chamber, an active valve, an electrode chamber to provide 66 °C of heat, glass slide as the substrate and PDMS chip. (**ii**) Photograph of the chip with two parallel microfluidic channels with capillary tubes to connect the chip to the syringe pump. (**iii**) Micrograph of the chip. (Reproduced with permission from [157]). (**C**) (**i**) Schematic illustration of the microfluidic chip to capture bacteria by inertial focusing. (**ii**) Dean vortices in action in a channel with trapezoid cross-section. (**iii**) Photograph of the 3D printed device. (Reproduced with permission from [158].

Purification and enrichment of bacteria from collected samples is crucial to accurately identify the causative organism. Microfluidic chips can be used for rapid purification and enrichment of microbes from clinical samples using different physical, chemical, and biochemical methods [128]. Using the principles of inertial differentiation, size differences, membrane filtration, acoustic separation, and different channel designs for margination methods, researchers have already explored microfluidic chips for bacterial detection [136,137,138,139] (Figure 6). Microorganisms can also be separated using chemical and biochemical methods in microfluidics, such as affinity-based captures to separate targeted bacteria from particles with similar densities and size [140,141]. The bacterial cells can also be lysed for on-chip DNA purification to perform subsequent molecular analysis like PCR in the same chip [142,143,144]. The chips allow control of the temperature and the introduction of primers and PCR reagents [128]. Plug-based droplet microfluidics confines individual bacteria into nanoliter droplets for rapid detection of bacteria and tests the antibiotic sensitivity. Some groups have already performed LAMP on the chips to detect bacteria in clinical samples with high sensitivity and in a shorter duration and simpler manner [145,146]. Jin et al. developed a chip capable of multiplex nucleic acid amplification and integrated it with LAMP for rapid detection of antibiotic resistance genes in lactic acid bacteria [147]. Researchers also have couple microfluidic chips with mass and fluorescence spectrometry for capturing, observing, and identifying multiple bacterial species along with screening multiple drugs [148,149]. By integrating various channels designs and patterns to create a steady antibiotic concentration gradient, bacterial antibiotic susceptibility and combinatorial effect of antibiotics at different doses can easily be tested in a single microfluidic chip [149].

Recently, microfluidic organ-on-chips (OOCs) have been gaining significant attention from research scientists and pharmaceutical companies for preclinical drug testing owing to the limitations of existing cell culture models and animal models [150]. OOCs are miniaturized cell culture devices with perfused channels that mimic the organ-level microenvironment and physiology [151]. Numerous research groups have already used these devices to fabricate organ-specific models for studying disease pathophysiology, host-pathogen interface, drug testing and toxicological analysis [151,152,153]. OOCs replicate the in vivo complex human tissue structure and the tissue-tissue interface, making it suitable for in vitro drug studies with physiologically relevant human responses [150]. Furthermore, biomarkers or target metabolites can be identified and quantified, which is a crucial step in the drug development process to bring out successful drug candidates into clinical trials [154]. The use of patient-derived primary cells, stem cells along with immune cells in the OOCs have the potential to fabricate patient-specific or population-specific chips, which can be applied towards personalized medicine applications and study the underlying disease pathology [155]. The possibility to create a “body-on-a-chip” by interconnecting two or more OOC devices will further assist the reproduction of the in vivo pharmacokinetics (absorption, distribution, metabolism, and excretion) and pharmacodynamics profiles of tested drugs and the multi/interorgan interactions and toxicity [156]. This will ultimately facilitate the identification of the most effective and safest drug, dosing, and combination to foster patient care. The efficacy, high-throughput, cost-effectiveness, and reproducibility of the microfluidic systems coupled with the advanced biosensors and diverse analytical methods can be applied for rapid detection, and analysis of pathogens, biomarker detection and screening of drugs for rapid diagnosis and effective treatment of patients with infectious diseases, preventing antimicrobial resistance.

## 6. Conclusions

Multidrug resistance has been a threat to clinicians and scientists over the past decades, as it entirely invalidates the theory and mechanisms of antibiotics regarding their therapeutic efficacy against the full range of microbes. Moreover, the modifications and adaptions in the development of microbes cause resistance against various therapeutics. Such complications and hurdles in the treatment of various infections brought the scientific community to a platform where the evolution of nanotechnology played a crucial role in overcoming the resistance of microbes. However, the evolution led to the emergence of various novel drug delivery systems, specifically nano-based drug delivery systems. These advancements by employing nanotechnology overcome microbial resistance of antibiotics by distinct mechanisms. In addition, the mechanisms of these NPs are interacting with the bacterial cell wall, generating reactive oxygen species, and inhibiting biofilm formation. Moreover, the literature has shown significant improvement in antimicrobial therapy with nanotechnology as a novel approach. However, several gaps need to be considered while designing any such approach for overcoming multidrug resistance, which eventually strengthens the translation process from bench-to-beside.

## Figures and Tables

**Table 1:** Application of advanced delivery systems for the superior therapeutic efficacy against various microorganisms.

SI. No	Nanoparticles	Microorganism Targeted	Inference
1	Solid lipid nanoparticles	*B. abortus*; *S. aureus*	- Increased encapsulation - Prolonged drug release - Enhanced drug penetration - Improve drug diffusion into the bacterial cell
2	Liposomes	*Klebsiella pneumoniae*; *Pseudomonas aeruginosa pneumonia*; *Methicillin-resistant Staphylococcus aureus*	- Increased antimicrobial activity by drug - High encapsulation efficiency - Enhanced drug release - Enhanced antimicrobial effect
3	Polymeric nanoparticles	*Candida albicans*; *Aspergillus niger*; *Bacillus subtilis Fusarium solani*; *Staphylococcus epidermidis*	- Bacterial cell disruption - Increased encapsulation efficiency - Prolonged antibacterial activity - Eliminating residual cell renewal - Elongated drug release.
4	Dendrimers	Gram-positive; Gram-negative	- Improve drug solubility - Enhanced antibacterial activity.
5	Inorganic nanoparticles i.Ag-NPs ii.Au-NPs iii.ZnO NPs iv.TiO_2_ NPs	*E. coli* *Staphylococcus aureus*, *Pseudomonas aeruginosa*, *Escherichia coli*, *Aspergillus fumigates*, and *Aspergillus niger* *Gram positive*; *Gram-negative bacteria* *Candida albicans* *Staphylococcus aureus. Candida albicans*	- Ag+ ion generation - Disruption of cell walls and electron - Transporters—DNA damage - Fall in ATP level - Preventing tRNA binding to ribosomes - Cell membrane rupture; Oxidative stress; Release of Zn+ ions - ROS production - Oxidative stress caused by ROS production - Site-specific DNA damage - Efficiently suppress the biofilm formation
6	NO-NPs	*Pseudomonas aeruginosa*; *Burkholderia cepacian*; *Staphylococcus aureus*; *Methicillin-resistant S. aureus*	- NO covalently binds DNA, proteins, and lipids at high concentrations, inhibiting or killing target microorganisms - NO release
